# COVID-19 Lockdown 2020 Changed Patterns of Alcohol and Cannabis Use in Swiss Elite Athletes and Bodybuilders: Results From an Online Survey

**DOI:** 10.3389/fspor.2021.759335

**Published:** 2021-11-16

**Authors:** Christian Imboden, Malte Christian Claussen, Samuel Iff, Boris B. Quednow, Erich Seifritz, Jörg Spörri, Johannes Scherr, Stefan Fröhlich

**Affiliations:** ^1^Private Clinic Wyss, Muenchenbuchsee, Switzerland; ^2^Department of Psychiatry, Psychotherapy and Psychosomatics, Psychiatric Hospital of the University of Zurich, Zurich, Switzerland; ^3^Psychiatric Services Grisons, Chur, Switzerland; ^4^Department of Psychiatry, Psychotherapy and Psychosomatics, Experimental and Clinical Pharmacopsychology, Psychiatric Hospital of the University of Zurich, Zurich, Switzerland; ^5^University Centre for Prevention and Sports Medicine, Department of Orthopaedics, Balgrist University Hospital, University of Zurich, Zurich, Switzerland; ^6^Sports Medical Research Group, Department of Orthopaedics, Balgrist University Hospital, University of Zurich, Zurich, Switzerland

**Keywords:** competitive sport, sports medicine, sports psychiatry, substance use, mental health

## Abstract

**Objectives:** During the COVID-19 pandemic, increased patterns of substance use have been reported in the general population. However, whether this also applies to athletes is not yet clear. This study aimed to detect changes in alcohol consumption and cannabis use in elite athletes and bodybuilders during the first COVID-19 lockdown in Switzerland.

**Methods:** Between April 25 and May 25, 2020, a cross-sectional online survey was conducted among bodybuilders and Swiss elite athletes who were active in Olympic sports and disciplines approved by the International Olympic Committee (IOC) on at least the national level. The collected data included information on alcohol and cannabis use during the last month (lockdown) and in the year before COVID-19 lockdown (pre-lockdown), daily training times, existential fears on a scale from 1 to 100, Patient Health Questionnaire-9 for depression (PHQ-9), Insomnia Severity Index (ISI), and State-Trait Anxiety Inventory (STAI).

**Results:** *N* = 275 athletes (elite athletes: *n* = 193; bodybuilders: *n* = 82) was included in this study. Both pre-lockdown and during lockdown, more bodybuilders used cannabis (both time points: *p* < 0.001) than elite athletes, and more elite athletes drank alcohol (pre-lockdown: *p* = 0.005, lockdown: *p* = 0.002) compared to bodybuilders. During lockdown, fewer athletes drank alcohol compared to before, but those who continued drinking did so on more days per week (*p* < 0.001, Eta^2^ = 0.13). Elite athletes were more likely to increase their drinking with 17.7 vs. 8.2% in bodybuilders. When compared to pre-lockdown measures, the number of athletes using cannabis did not change during lockdown. Only three of 203 elite athletes reported using cannabis during lockdown; this contrasts with 16 of 85 bodybuilders. In a multivariate regression model, existential fears and a lower ISI score were significant predictors for increased alcohol consumption during the lockdown in the entire sample. In a model based on elite athletes only, male sex and a lower ISI score predicted increased alcohol consumption. In a bodybuilder-based model, predictors of increased alcohol consumption were existential fears and trait anxiety.

**Conclusion:** We suggest identifying athletes who are at risk for increased alcohol and cannabis use; we suggest this to be able to professionally support them during stressful times, such as the COVID-19 pandemic.

## Introduction

In spring 2020, during the first wave of the 2019 coronavirus disease (COVID-19) pandemic, athletes all over the world faced substantial challenges (Claussen et al., 2020). For example, training opportunities were impaired, and many major sporting events were canceled or carried out without spectators, leading to financial burdens for many sports clubs. Even the 2020 Olympic summer games, for which many athletes had trained for years, were postponed; this disrupted the 4-year structure Olympic athletes were used to and led to increased stress and professional uncertainty (Håkansson et al., 2020b).

In Switzerland, a national emergency was declared in mid-March 2020, leading to the first lockdown that lasted from March 17 to May 10, 2020. This lockdown included travel bans, the prohibition of all large-scale events, restrictions on public gatherings (including sports competitions), a general stay-at-home recommendation, and the closing of all sports facilities. Private training was still possible, and no curfew was imposed. Therefore, most elite athletes and bodybuilders had to drastically adapt their lifestyle and training patterns, as social contacts had to be limited and they were confined to home in terms of training. The closing of training centers and cancellations of competitions were described as the most disruptive issues for athletes during the COVID-19 pandemic (Parm et al., 2021). To cope with this critical phase, it was recommended that athletes maintain a routine, continue with healthy eating and sleeping patterns, try new forms of training at home, keep in touch with peers, and reduce the consumption of information from unreliable media (Claussen et al., 2020; Tayech et al., 2020).

Considering the fact that athletes are affected by mental health disorders at least as much as the general population (Gouttebarge et al., 2019; Reardon et al., 2019) and knowing about the massive challenges related to COVID-19 restrictions we've described, special attention should also be paid to mental health changes in athletes during the COVID-19 pandemic. Gouttebarge et al. (2020) studied a sample of professional football players (*n* = 1,602) and found an increase in anxiety and depression during the lockdown period. Increased anxiety has also been reported by other authors in Estonian (Parm et al., 2021) and Brazilian (Soares et al., 2021) samples of athletes. On the other hand, a Turkish study found that the mental health of professional athletes was better during the lockdown than in a matched group of non-athletes, suggesting that regular physical activity has a protective effect (Senişik et al., 2020). A systematic review of 14 studies reported a reduction in training in the majority of athletes and a significant increase in negative emotions during the COVID-19 pandemic (Jurecka et al., 2021).

During the lockdown of spring 2020, an increase in alcohol consumption was reported in 16–25% of the general population (Szajnoga et al., 2020; Garnett et al., 2021; Jacob et al., 2021). However, a larger proportion of the general population reported a reduction in their drinking frequency (Szajnoga et al., 2020; Bollen et al., 2021). Increased drinking has been associated with young age (18–24 years), male sex, more depressive symptoms, and lower mental well-being (Szajnoga et al., 2020; Jacob et al., 2021). In a web-based survey conducted among Swedish elite athletes from team sports (*n* = 327), 16% of respondents reported that they “drink more,” and 13% reported that they “drink less” than before the COVID-19 pandemic (Håkansson et al., 2020a). During the summer of 2020, Shaw and coworkers Shaw, Bertrand (Shaw et al., 2021) studied a sample of 32 master cyclists (≥35 years of age) and found a significant increase in alcohol consumption compared to before the pandemic (5-fold in men and 1.5-fold in women). However, to the best of our knowledge, there has been no further published data on changes in alcohol consumption among elite athletes during the COVID-19 pandemic.

Bodybuilders often have similar training schedules to elite athletes. However, instead of athletic performance, bodybuilder training is mainly aimed at gaining muscle size and improving body image; therefore, it often includes highly specialized eating regimes (Pickett et al., 2005). Since most bodybuilders are not regularly subjected to doping controls, the use of illicit substances, such as cannabis, may be more widespread than among elite athletes. The use of prohibited substances, especially anabolic-androgenic steroids (AAS), is also higher among bodybuilders than among other athletes (Van Eenoo and Delbeke, 2003). Simultaneously, athletes using AAS are at increased risk of using illicit substances, such as cannabis and stimulants (Gårevik and Rane, 2010). Data on alcohol consumption among bodybuilders is extremely scarce. In a Brazilian sample, 118 of 145 recreational bodybuilders (81%) were drinking alcohol (Schwingel et al., 2014), while a study from the Netherlands found that 70% of bodybuilders using AAS concomitantly drink alcohol and 23% consume cannabis. We found no other publications on alcohol and/or cannabis use in bodybuilders. There is no data known to us on the impact of the COVID-19 lockdown on bodybuilders. Cannabis is widely used in the general population of Switzerland and has a lifetime prevalence of 28.3%, which is a number that is expected to increase further in the following years (Vogel et al., 2019). Among elite athletes, data on cannabis use is heterogeneous across samples, with up to 25% of athletes having used cannabis in the last year (Docter et al., 2020). Cannabis is estimated to have replaced tobacco as the second most commonly used drug among athletes, with alcohol being the most commonly used drug (Brisola-Santos et al., 2016). However, cannabis is still used less among elite athletes than in the general population (McDuff et al., 2019). Like the data on alcohol use, many studies on cannabis consumption among athletes have been conducted in samples of college athletes. To the best of our knowledge, there is no data on the changes in alcohol and cannabis use among athletes during the COVID-19 lockdown.

Accordingly, the purpose of this study was to: (1) compare Swiss elite athletes and bodybuilders in terms of their substance use during Switzerland's first COVID-19 lockdown and (2) assess changes in alcohol and cannabis use due to the COVID-19 lockdown. We hypothesized that both groups would increase their alcohol and cannabis use, and that participants with lower mental health variables and higher existential fears would be more likely to increase drinking and cannabis consumption.

## Materials and Methods

### Design and Study Population

During the first wave of the COVID-19 pandemic, a cross-sectional REDCap-based online survey (REDCap 9.10.0^©^ 2020 Vanderbilt University) was conducted among elite athletes and bodybuilders. The online questionnaire was sent to (1) athletes in Switzerland who performed in Olympic sports or IOC-approved disciplines on at least a national level and (2) bodybuilders. For team sports, questionnaires were distributed by the sports clubs, while questionnaires for individual sports were distributed by the respective national sports federations. For bodybuilders, the questionnaires were distributed by peers in our network. In order to reach a high proportion of Swiss athletes, the questionnaire was available in both German and French. Data collection took place from April 25 to May 25, 2020.

Respondents were included in the study if they (1) trained at least 1 h per day pre-lockdown, (2) were 18 years or older, and (3) performed in Olympic disciplines or sports recognized by the IOC (elite athletes) or were bodybuilders. There was no financial or other compensation for participation in this study.

### Ethical Approval

The protocol underlying this study was reviewed by the local ethics committee (KEK-ZH-NR: Req-2020-00408) and was judged not to fall under the scope of the Human Research Act (HRA).

### Data Collection and Evaluation

Besides demographic data, including age, gender, and type of sport, we collected information about alcohol drinking and cannabis consumption before and during the lockdown. To determine which members of our sample were full-time athletes, we queried whether their income earned from participation in sports is sufficient to cover their expenses for daily living. If a participant was able to cover their daily expenses with their income earned from sports, they were considered a full-time athlete.

First, respondents had to recall whether they drank alcohol or consumed cannabis at all during the prior month (lockdown) and in the year before lockdown (pre-lockdown). Second, they were asked how many days per month they consumed these substances during the two different periods. Recall of prior alcohol use during a given period has been used in previous studies (Bollen et al., 2021) and has been found to be a reliable method (Ekholm et al., 2008). However, other studies queried alcohol intake during a typical week (Bollen et al., 2021). Since we expected low drinking frequencies in our sample, we deviated from this method and asked for participants to report the number of drinking days during a typical month. Additionally, participants were asked if they experienced existential fears during lockdown, and this information was reported on a visual analog scale from 0 to 100 (“I have no existential fears”—“I experience very strong existential fears”). Training load pre-lockdown and during lockdown (hours per day) were also surveyed. All questions are provided in German with English translation as Supplementary Material. To measure mental health variables, the Patient Health Questionnaire-9 for depression (PHQ-9), (Arroll et al., 2010), Insomnia Severity Index (ISI), (Bastien et al., 2001), and State-Trait Anxiety Inventory (STAI), (Spielberger, 1983) were obtained from all respondents.

### Statistical Analyses

Descriptive variables were presented as mean value and standard deviation (SD) for all athletes and for elite athletes and bodybuilders separately. Differences between groups were calculated by Chi^2^ for categorical variables and analysis of variance (ANOVA) for continuous variables. For changes between pre-lockdown and lockdown measures, the McNemar test was performed for categorical variables (use/no use). For changes in frequency (days/month), paired *t*-tests were calculated. To assess individual increase in drinking frequency, a new variable called “increase in alcohol use” was defined; when athletes used alcohol on at least one day more per month during lockdown compared to pre-lockdown rates, they were determined to have experienced an increase in alcohol use. To identify factors associated with increased frequency of alcohol consumption, we performed multiple linear regression analysis with the dependent variable “alcohol use during lockdown” (days/month), and we used the independent factors of gender, team-sports (not for bodybuilders), existential fears during lockdown, state-and trait-anxiety, ISI-sum-score, and PHQ-9 as predictors. Regression models were calculated for the entire sample and for elite athletes and bodybuilders separately. Since the metrics of the independent variables were highly heterogeneous, standardized coefficients (Beta) were reported. Due to low numbers of cannabis use, no regression analysis was calculated for “cannabis use during lockdown.” Only respondents with valid data on substance use during lockdown were included in the analysis. The level of significance was set at *p* < 0.05. All analyses were carried out using SPSS Version 24.

## Results

### Sample Characteristics

A sample of 288 Swiss athletes (elite athletes: 203; bodybuilders: 85) answered the questionnaire. Thirteen respondents gave no answers on substance use and were excluded from the study. 275 athletes were included in the final analysis (82, 29.8% bodybuilders; 193, 70.2% elite athletes). Of the group of elite athletes, 99 (51.3%) performed in summer sports and 94 (48.7%) in winter sports. Significantly more athletes in Olympic or IOC-recognized sports earned enough income through sports to support their lives (52.3%) when compared to bodybuilders (30.5%; *p* = 0.001). Thus, the sample consists of a large proportion of professional elite athletes. The bodybuilder subsample consisted of significantly more men (87.8%) than the elite athletes (54.9%; *p* < 0.001), and their training volume was significantly lower at both time points (pre-lockdown: *p* < 0.001; lockdown: *p* < 0.001). Pre-lockdown elite athletes reported daily training volumes from 1 to 7 hours and bodybuilders from 1 to 4 hours. Bodybuilders showed significantly higher ISI scores (*p* < 0.001), higher depressive symptoms (*p* < 0.001), and anxiety symptoms (state anxiety: *p* < 0.001, trait anxiety: *p* < 0.001), as can be seen in detail in Table 1. All athletes significantly decreased their daily training times during lockdown, but the effect was larger in bodybuilders (*t*_(81)_ = 5.743, *p* < 0.001) than in elite athletes (*t*_(192)_ = 4.140, *p* < 0.001).

**Table 1:** Sample description.

**Sample description**				
	**Bodybuilding**	**Elite athletes**	***p***	**All athletes**
***N*** **(%)**	82	193		275
	(29.8%)	(70.2%)		(100.0%)
**Sports** ***(n)*****:**				
Summer sports		99		
		(51.3%)		
Winter sports		94		
		(48.7%)		
Team sports		59		
		(30.6%)		
**Age (yrs)**	26.54	24.08	0.002	24.82
	(7.34)	(5.21)		(6.02)
**Gender (*****n*****)**:				
Males	72	106	<0.001	178
	(87.8%)	(54.9%)		(61.8%)
Females	10	87		97
	(12.2%)	(45.1%)		(33.7%)
**Additional characteristics:**				
Training pre-lockdown (hours/day)	2.28	3.14	<0.001	2.882
	(0.80)	(1.39)		(1.30)
Training lockdown (hours/day)	1.61^*^	2.70^*^	<0.001	2.37^*^
	(0.90)	(1.22)		(1.24)
Sufficient income from sports (#yes)	25	101	0.001	126
	(30.5%)	(52.3%)		(43.8%)
Existential fears (0–100)	21.7	22.2	0.779	21.9
	(28.20)	(26.68)		(27.10)
ISI score (0–28)	9.1	5.4	<0.001	6.5
	(5.93)	(4.11)		(4.98)
PHQ-9 score (0–27)	9.4	4.5	<0.001	5.9
	(5.51)	(3.16)		(4.56)
State anxiety (20–80)	37.4	29.2	<0.001	31.5
	(13.34)	(11.41)		(12.51)
Trait anxiety (20–80)	35.4	27.2	<0.001	29.6
	(11.50)	(10.98)		(11.72)

*Means (SD) or N (%); Olympic sports include n = 105 summer sports and n = 98 winter sports. Means; P-values for differences between groups (bodybuilders, elite athletes): Chi^2^ for categorical variables, analysis of variance (ANOVA) for continuous variables*.

* *p < 0.001 vs. pre-lockdown (paired t-test). SD, standard deviation; ISI, Insomnia Severity Index; PHQ-9, Patient Health Questionnaire-9*.

### Alcohol and Cannabis Use During Lockdown

When comparing their pre-lockdown rates, significantly more bodybuilders used cannabis (*p* < 0.001) and significantly more elite athletes drank alcohol (*p* = 0.005). As shown in Table 2, significantly fewer athletes (entire sample: *p* = 0.001; elite athletes only: *p* = 0.008) continued drinking alcohol during lockdown, but there was no significant change for bodybuilders (*p* = 0.078). Cannabis was used by fewer athletes during lockdown, but the change did not reach statistical significance (entire sample: *p* = 0.424). Those athletes who continued drinking alcohol did so on significantly more days per month (*p* < 0.001). Elite athletes were more likely to increase their alcohol consumption: compared to 8.2% of bodybuilders, 17.7% of elite athletes reported drinking on more days per month during lockdown than before. The 19 athletes who continued cannabis use during lockdown also reported significantly increased frequency (entire sample: *p* = 0.049). Bodybuilders were significantly more likely to use cannabis during lockdown than elite athletes (*p* < 0.001) and less likely to drink alcohol (*p* = 0.002).

**Table 2:** Alcohol and cannabis use.

	**Pre-lockdown**	**Lockdown**	***t***	***p***
***All athletes: n = 275***				
Alcohol use (yes)	160	133		0.001
	(58.2%)	(48.4%)		
Alcohol frequency (days/month)	2.84	5.12	−4.23	<0.001
	(0.03)	(0.04)		
Increased alcohol frequency during lockdown (yes)		43		
		(14.9%)		
Cannabis use (yes)	23	19		0.424
	(8.4%)	(6.9%)		
Cannabis frequency (days/month)	2.57	4.53	−2.18	0.049
	(4.18)	(4.74)		
***Elite athletes only: n = 193***				
Alcohol use (yes)	123^*^	105^*^		0.008
	(63.7%)	(54.4%)		
Alcohol frequency (days/month)	3.19	5.43	−3.761	<0.001
	(3.27)	(4.64)		
Increased alcohol frequency during lockdown (yes)		36		
		(17.7%)		
Cannabis use (yes)	5^*^	3^*^		0.500
	(2.6%)	(1.6%)		
Cannabis frequency (days/month)	0.6	1.67		(**)
	(0.55)	(0.58)		
***Bodybuilders only: n = 82***				
Alcohol use (yes)	37^*^	28^*^		0.078
	(43.5%)	(32.9%)		
Alcohol frequency (days/month)	1.68	3.96	−2.561	0.018
	(1.40)	(3.29)		
Increased alcohol frequency during lockdown (yes)		7		
		(8.2%)		
Cannabis use (yes)	18^*^	16^*^		0.774
	(21.2%)	(18.8%)		
Cannabis frequency (days/month)	3.11	5.06	−1.969	0.077
	(4.59)	(5.00)		

*Means, (SD) or N (%);*

(*) *, Chi^2^ p < 0.01 between elite athletes and bodybuilders;*

(**) *, no statistics for cannabis due to anecdotal numbers; P-values: Use (yes/no), McNemar test; Frequencies (days/month), paired t-test. SD, standard deviation*.

### Multivariate Regression

In the linear regression model of the entire sample (*R*^2^ = 0.11, df = 7, *F* = 2.154, *p* = 0.043), significant predictors for alcohol frequency (days/month) were “existential fears during lockdown” and better sleep (which is reflected by a lower ISI score). In the regression model of the subsample of elite athletes (*R*^2^ = 0.13, df = 7, *F* = 2.090, *p* = 0.052), male sex and lower ISI score were revealed as significant predictors for increased alcohol consumption. In the regression model of the bodybuilder subsample (*R*^2^ = 0.56, df = 6, *F* = 4.075, *p* = 0.009), predictions for increased alcohol consumption were strongest and included “existential fears during lockdown” and trait anxiety. Detailed results are summarized in Table 3. Due to the small number of athletes using cannabis during lockdown, we did not calculate regression models for cannabis use.

**Table 3:** Linear regression results for alcohol frequency during lockdown (days/month).

**Sample**	**All athletes**	**Elite athletes only**	**Bodybuilders only**
Alcohol consumption before (yes)	0.031	0.080	−0.135
	*(0.752)*	*(0.432)*	*(0.586)*
Male sex	0.150	0.240^*^	−0.029
	*(0.095)*	(0.021)^*^	*(0.873)*
Existential fears during lockdown	0.213^*^	0.079	0.727^*^
	(0.034)^*^	*(0.475)*	(0.013)^*^
ISI sum-score	–0.237^*^	–0.219^*^	−0.054
	(0.031)^*^	(0.039)^*^	*(0.843)*
PHQ-9 sum-score	0.054	0.088	0.080
	*(0.706)*	*(0.537)*	*(0.819)*
Trait anxiety	0.054	0.086	0.582^*^
	*(0.695)*	*(0.624)*	(0.016)^*^
State anxiety	0.065	0.067	−0.485
	*(0.676)*	*(0.678)*	*(0.137)*
Team sports	−0.062	−0.175	–
	*(0.483)*	*(0.105)*	–

*Standardized regression coefficient Beta and (p-value);*

* *p < 0.05. Multivariate linear forward regression. ISI, Insomnia Severity Index; PHQ-9, Patient Health Questionnaire-9*.

## Discussion

This study found that, during lockdown, fewer athletes consumed alcohol than before, but those who continued drinking did so on more days per month. A meaningful proportion of athletes (entire sample: 14.9%; elite athletes only: 17.7%) increased their alcohol consumption. In the full sample, higher existential fears and better sleep were found to be predictors of increased alcohol consumption, while male sex and better sleep were found to be predictors in elite athletes. Fewer bodybuilders increased their alcohol consumption (8.2%), and group-specific regression analysis revealed that existential fears and trait anxiety are more likely to be the main predictors of increased alcohol consumption in the bodybuilder subsample. The proportion of elite athletes who increased their drinking is very similar to the findings of Håkansson et al. (2020a), who reported increased alcohol consumption in 16% of Swedish athletes during lockdown. Moreover, that male sex is a predictor for increased alcohol consumption is a well-known fact in the general population (Erol and Karpyak, 2015) and among athletes (Souter et al., 2018). Our finding that an increase in drinking is more likely to be seen in male elite athletes during COVID-19 lockdown has also been reported previously (Shaw et al., 2021).

Alcohol is commonly believed to be associated with poor sleep and has been shown to have sleep-promoting effects but disrupting sleep architecture (He et al., 2019). Given this, the association of better sleep scores and increased alcohol consumption seems surprising. However, the frequency of alcohol consumption in our sample is rather low, and we did not collect data on the number of drinks per occasion. Because of this, the negative effects of alcohol on sleep architecture might have been marginal; this would explain why, on some nights, athletes who normally drink only small quantities might actually benefit from reduced sleep latency due to alcohol consumption.

In the entire sample, there is an association of existential fears with drinking frequencies that is consistent with our hypotheses. However, this association is mainly driven by the bodybuilder sample. An increase in alcohol consumption has already been reported during the COVID-19 pandemic for the general population and has been associated with more time, boredom, and the negative effects the pandemic has had on employment (Weerakoon et al., 2021). Since bodybuilders show higher state and trait anxiety than elite athletes, anxiety might play a more important role in motivating them to drink alcohol. They also train fewer hours per day and may, therefore, have more time to worry; this could be a reason why vulnerable bodybuilders drink more.

Those athletes who continued using cannabis during lockdown did so more often. However, only a small number of elite athletes used cannabis at all. It has been previously described that lower rates of cannabis use are found among athletes than in the general population (McDuff et al., 2019); this may be due, at least in part, to higher awareness of national and international doping-regulations, especially among elite athletes. This might apply less to bodybuilders because they are less likely to be subject to doping controls. Additionally, due to their greater tendency to restrict their eating and to have concerns about body fat, the high caloric density of alcohol might be less attractive for bodybuilders (Pickett et al., 2005). This might explain our findings that bodybuilders were significantly more likely to use cannabis and less likely to drink alcohol when compared to elite athletes.

The restrictions of the lockdown during the pandemic had a strong impact on elite athletes by inducing higher stress levels and, consequently, increasing symptoms of anxiety and depression (Gouttebarge et al., 2020; Pillay et al., 2020). To the best of our knowledge, there is no comparable data on bodybuilders. As our data suggests, however, only a certain proportion of athletes increased their drinking frequency. Therefore, we consider it important to identify those athletes who tend to increase drinking during stressful times in order to be able to support them psychologically. To better understand the psychosocial situations and needs of individual athletes during stressful times, we should focus on athletes' mental health. Ideally, this would include making use of structured evaluations like those proposed by the International Olympic Committee (Reardon et al., 2019), the International Society for Sports Psychiatry (ISSP), (Reardon and Factor, 2010), or the Swiss Society for Sports Psychiatry and Psychotherapy (SSSPP), (Claussen et al., 2019). As suggested by Mehrsafar et al. (2020), health authorities and sports communities should, therefore, aim at maintaining athletes' mental health and athletic activities during a crisis like the COVID-19 pandemic.

Our study provides data on a considerable sample of Swiss elite athletes, with more than half of them being able to make a living from sports. To date, this is the only study that has assessed Swiss athletes and bodybuilders for substance use during the COVID-19 pandemic. Moreover, data on alcohol consumption in athletes is still scarce and has so far mainly focused on data from US college athletes (McDuff et al., 2019). However, despite providing important new insight into the field, our study has several limitations that one should be aware of when interpreting the study findings: (1) As we conducted a web-based survey, and the recruited sample may not be entirely representative of elite athletes and bodybuilders around the world, limited external validity and selection bias may have occurred. Since data on alcohol and cannabis use is sensitive information, data quality must also be questioned (e.g., for information bias). Since cannabis is a prohibited substance in competitions, this especially applies to cannabis use among elite athletes. (2) Data concerning substance use was assessed retrospectively from memory for two different periods and, therefore, may have suffered from significant recall bias. (3) Since data was collected by questionnaire, there is the possibility of subjective underreporting of substance use. (4) We did not measure the length of time athletes have been involved in their training schedules. Athletes (especially bodybuilders who took up intensive training routines shortly before the COVID-19 pandemic) could add to the heterogeneity of our sample. (5) Retrospectively, we should have assessed the number of standard drinks per occasion. Therefore, we have no information on possible binge drinking, and our data must be interpreted with caution. (6) Due to our inclusion criteria (i.e., at least one hour of training per day), the sample is mixed: it includes professional and amateur level athletes for both subsamples. Additional information (e.g., about whether participants hold a Swiss Olympic card) would have increased our sample description. On the other hand, McKinney et al. (2019) propose a definition of elite athletes if weekly training consists of ≥10 h and competitive athletes for 6 to 10 h. Following their proposition, our sample consists of a large proportion of elite athletes. Concerning the methodology of our study, it must be mentioned that there are serious limitations to comparing data from two prior periods of different duration (pre-lockdown: 1 year, and lockdown: 1 month) assessed by a cross-sectional design, and these comparisons might be significantly biased. Therefore, our reported changes over time must be interpreted with caution.

## Conclusion

During the first COVID-19 lockdown in Switzerland, a small but substantial number of elite athletes and bodybuilders increased their consumption of alcohol and cannabis. Concerning the two substances, bodybuilders were more likely to consume cannabis than elite athletes, who, in turn, were more likely to drink alcohol. Existential fears and better sleep were significant predictors for increased drinking in the entire sample, while male gender was found to be a significant predictor for elite athletes, and anxiety was found to be a significant predictor only for bodybuilders. However, the mean frequency of alcohol and cannabis use remained rather low. Nonetheless, we suggest better identifying those athletes at risk for increased drinking; this would enable us to better support them during stressful times, such as during the COVID-19 pandemic.

## Data Availability Statement

The raw data supporting the conclusions of this article will be made available by the authors, without undue reservation.

## Ethics Statement

The studies involving human participants were reviewed and approved by Kantonale Ethikkommission Zürich (KEK-ZH-NR: Req-2020-00408). Written informed consent for participation was not required for this study in accordance with the national legislation and the institutional requirements.

## Author Contributions

All authors contributed to conception and design of the study. MC and JS (6th author) organized the database. CI, MC, and SI performed statistical analysis. CI and MC wrote the first draft of the manuscript. All authors contributed substantially to the final manuscript revision and read and approved the submitted version.

## Conflict of Interest

The authors declare that the research was conducted in the absence of any commercial or financial relationships that could be construed as a potential conflict of interest.

## Publisher's Note

All claims expressed in this article are solely those of the authors and do not necessarily represent those of their affiliated organizations, or those of the publisher, the editors and the reviewers. Any product that may be evaluated in this article, or claim that may be made by its manufacturer, is not guaranteed or endorsed by the publisher.
