# Supplementary material for: COVID-19 Lockdown 2020 Changed Patterns of Alcohol and Cannabis Use in Swiss Elite Athletes and Bodybuilders: Results From an Online Survey
Source: Front Sports Act Living. 2021 Nov 16;3:759335. doi: 10.3389/fspor.2021.759335 (PMC8635023; doi:10.3389/fspor.2021.759335)
Supplement: Supplementary file 1 [file Table_1.docx]

**Supplemental Material**

Questions of online survey (besides standardized questionnaires)

Questions were sent to athletes in German or French.

| **German (original)** | **English translation** |  |
| --- | --- | --- |
| Geburtsjahr | Year of birth | year |
| Biologisches Geschlecht | Biological sex | male/female |
|  | | |
| Hatten Sie bis zum Beginn der COVID-19-Pandemie ausreichend Einkünfte für den Lebensunterhalt aus dem Leistungssport? | Did your income from sports cover your expenses of daily living before the COVID-19 pandemic? | yes/no |
| Haben Sie aktuell finanzielle Existenzängste? | Do you currently suffer from financial existential fears? | 0-100 |
|  | | |
| Was ist Ihre Hauptsportart? | What is your main sport? | discipline |
| *Wie viele Stunden pro Tag wenden/wendeten Sie für Training und Wettkampf auf...* | *How many hours per day are spent with training or competition…* |  |
| - Aktuell (letzter Monat)? | - Currently (last month)? | hours/day |
| - Im Monat vor Beginn der COVID-19-Pandemie? | - During the month before the COVID-19 pandemic? | hours/day |
| ***Substance use*** | | |
| Haben Sie aktuell (im letzten Monat) Alkohol konsumiert? | Did you use alcohol during the last month? | yes/no |
| - Falls ja, an wie vielen Tagen? | - If yes, on how many days? | Days/month |
| Haben Sie im Jahr vor der COVID-19-Pandemie (ca. März19 bis März20) Alkohol konsumiert? | During the year before the COVID-19 pandemic (March19 to March20), did you use alcohol? | yes/no |
| - Falls ja, an wie vielen Tagen im Monat (durchschnittlich)? | - If yes, on how many days per month (average)? | Days/month |
|  | | |
| Haben Sie aktuell (im letzten Monat) Cannabis konsumiert? | Did you use cannabis during the last month? | yes/no |
| - Falls ja, an wie vielen Tagen? | - If yes, on how many days? | Days/month |
| Haben Sie im Jahr vor der COVID-19-Pandemie (ca. März19 bis März20) Cannabis konsumiert? | During the year before the COVID-19 pandemic (March19 to March20), did you use cannabis? | yes/no |
| - Falls ja, an wie vielen Tagen im Monat (durchschnittlich) | - If yes, on how many days per month (average)? | Days/month |
